# Mechanisms Behind Graphitization Modification in Polycrystalline Diamond by Nanosecond Pulsed Laser

**DOI:** 10.3390/ma17246200

**Published:** 2024-12-19

**Authors:** Xinrui Cui, Chunyu Zhang, Guo Li, Chengwei Song, Wentao Qin, Tao Wang

**Affiliations:** Laser Fusion Research Center, China Academy of Engineering Physics, Mianyang 621900, China; cxr18846439591@163.com (X.C.); liguo51404@163.com (G.L.); songchengwei@126.com (C.S.); wentaoqin9323@163.com (W.Q.); wang.tao_1981@163.com (T.W.)

**Keywords:** graphitization modification, ultraprecision machining, polycrystalline diamond, molecular dynamics (MD) simulation

## Abstract

The ultraprecision machining of diamond presents certain difficulties due to its extreme hardness. However, the graphitization modification can enhance its machinability. This work presents an investigation into the characteristics of the graphitization modification in polycrystalline diamond induced by a nanosecond pulsed laser. In this paper, the morphology of microgrooves under laser modification was observed, material deposition and graphitization in different regions were researched, and the regularities of microgrooves at different laser powers were obtained. A molecular dynamics (MD) simulation was carried out to reveal the mechanism behind graphitization modification; when the pulse laser acts on the diamond surface and the temperature rises to the critical temperature of graphitization, the graphite crystal nuclei form and grow, resulting in the graphitization modification. It was confirmed that the existence of grain boundaries (GBs) contributed to the graphitization of polycrystalline diamond during laser modification. It was predicted that a lower laser power could cause a higher proportion of graphitization. The results of ablation thresholds and the effect of the defocusing position on the graphitization of diamond showed that for a fixed laser power, the highest graphitization ratio could be obtained when the defocusing quantity was optimized. Finally, the results of precision grinding experiments verified the feasibility of using laser graphitization pretreatment to improve the efficiency and quality of precision grinding.

## 1. Introduction

Diamond, with its excellent mechanical hardness and optical properties [1], has been widely used in various elements [2], such as semiconductor components [3], cutting tools [4], dressing tools [5], and inertial confinement fusion (ICF) capsules [6,7]. For ICF experimental capsules, a defect-free surface is needed, and the shape and surface smoothness will directly affect the focusing and reaction efficiency of ignition experiments. However, processing polycrystalline diamond is difficult and inefficient due to its high hardness and excellent chemical stability. For the machining of diamond, methods have been extensively studied, such as mechanical polishing [8], ion beam etching [9], and chemical machining [10,11], and the results show that they are time-consuming. Therefore, a new method of surface modification is required to reduce the hardness of polycrystalline diamond and improve the machinability and quality of the diamond surface.

Currently, for the micromachining of the diamond surface, technology with pulsed lasers is showing potential [12,13]. The graphitization phase transition occurs in the diamond during laser ablation [14,15,16], and the hardness of graphite is lower than that of diamond. Graphitization modification represents a significant research direction in improving the machinability of diamond surface. Although laser-induced surface modification has been widely used in the field of auxiliary machining [17,18], mature methods in the field of diamond surface modification still remain to be elucidated. Thus, it is important to clarify the mechanisms and the influencing factors in laser graphitization. The results of numerical simulations for diamond indicate that the transition from diamond to graphite occurs along a variety of pathways, depending on the wavelength of the laser pulse [19]. The difference in resistance and thickness between different layers could be explained by a model of the formation of orientated nanocrystalline graphite [20]. Although graphitization during the pulsed laser processing of diamond has been extensively studied, there have been few attempts to investigate the specifics of the atomic evolution in the laser graphitization of diamond and the effect of laser power on the ratio of graphitization to material removal, which can shed light on the mechanism behind the laser-induced graphitization modification of diamond and enable us to control the process to achieve a modified surface that enhances the machinability of diamond; such investigations can be carried out by using molecular dynamics (MD) approaches. However, there has been very little published work on the MD simulation of diamond under laser ablation, and the studies are mostly based on the mechanical properties of diamond [21] or the process of material removal [22]. Therefore, more research efforts are still needed to investigate MD simulation and the underlying mechanisms behind the graphitization of diamond under laser pulses.

In this paper, the dynamic evolution of atoms from a diamond structure to a graphite structure is presented, and the effects of GBs and laser power on the laser graphitization of diamond are revealed by MD simulation. The morphology of different regions under laser modification was observed, and the graphitization was detected by Raman spectroscopy to study the mechanisms behind graphitization modification. To obtain the graphitized surface with optimized parameters, the ablation threshold under different pulse numbers was calculated and the effect of the laser power on graphitization of diamond was further validated by defocusing experiments. Finally, the modified surface and the unmodified surface were processed under the same grinding conditions, and the influence of laser modification pretreatment on the efficiency and quality of subsequent processing was studied.

## 2. Experimental Details and Simulation Model

### 2.1. Experimental Setup

**Figure 1:**
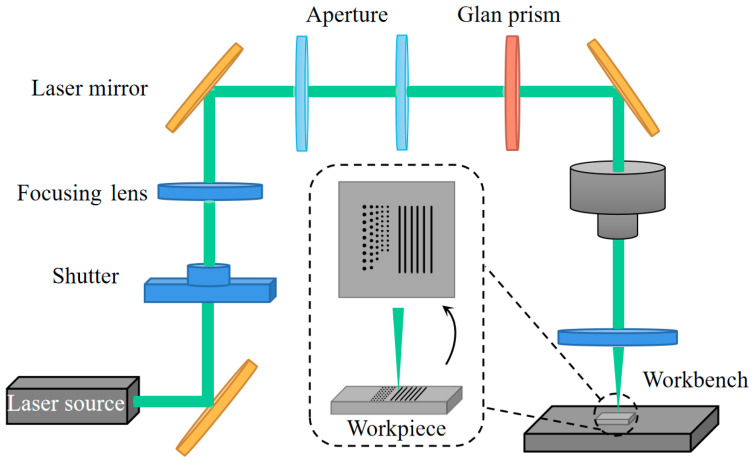
Experimental devices for nanosecond pulsed laser modification.

Figure 1 shows the laser system for nanosecond pulsed laser modification. The devices used in the experiments included a nanosecond pulsed laser source, a mechanical laser shutter, two apertures, two focusing lenses, a Glan prism, a charge-coupled device (CCD), and a number of laser mirrors. The nanosecond pulsed laser applied in the experiments had a repetition frequency of 5 kHz and a wavelength of 532 nm. The laser energy followed a Gaussian distribution. The laser power on the target surface varied from 50 mW up to 130 mW. A power dynamometer with 0.1 mW accuracy was used to measure the laser power. The surface morphology and ablation diameter produced by laser modification were examined with a scanning electron microscope (SEM) and optical microscopy. The alteration of the diamond surface before and after modification was characterized by Raman spectroscopy, with an integration time of 4 s and using a confocal setup with a 532 nm laser excitation source. Due to the challenges associated with measuring the absolute intensity in Raman spectroscopy, the normalized ID/IG intensity ratio has been predominantly utilized as a metric for assessing the extent of graphitization. The ratio of graphite intensity (IG) to diamond intensity (ID) was calculated by the following: (1) the baseline calibration of the Raman spectra, (2) multiple peak fitting (Gaussian fit), and (3) calculating the IG and ID (i.e., the area of the peak). The surface hardness of the diamond before and after laser modification was measured by a Vickers indentation instrument. To accurately measure the Vickers hardness of the surface modified at various scanning intervals, the hardness was measured at three distinct positions within the modified area under a specific load. The results from these three measurements were then averaged to minimize error.

The sample used in experiments is polycrystalline chemical vapor deposition (CVD) diamond, which has a roughness less than 15 nm and is polished on both sides. The dimensions of the specimen are 4 mm × 4 mm × 1 mm. To prevent the influence of dust and impurities on laser energy absorption, the sample was cleaned by ultrasonication before the experiments [23]. The nanosecond laser beam was transmitted from the light source to the focusing lens through the laser mirror and irradiated on the diamond surface in the vertical direction. Holes and grooves were machined on the sample workpiece using the nanosecond laser, as shown in Figure 1.

### 2.2. MD Simulation Model

**Figure 2:**
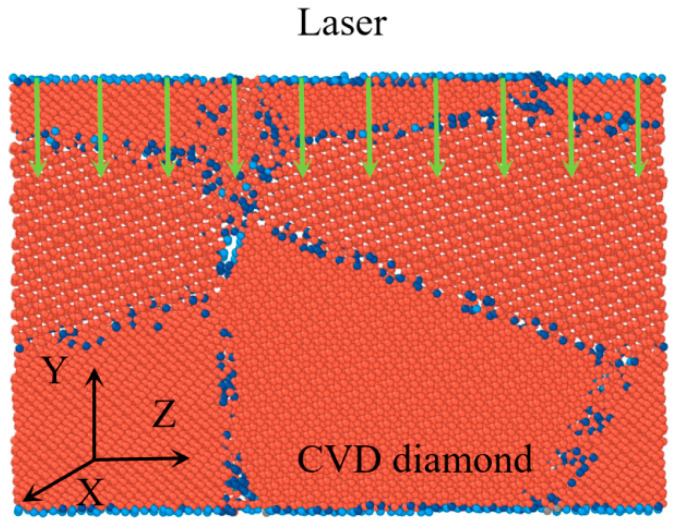
MD simulation model of the laser modification of CVD diamond.

This study investigates the evolution of diamond under laser modification using a model comprising a diamond specimen and a laser heat source, as shown in Figure 2. Considering the time cost and efficiency of calculation, the size of the model was greatly reduced compared with the actual experimental specimen. The model had dimensions of 12.0 nm, 8.0 nm, and 12.0 nm in the X, Y, and Z directions, respectively. Periodic boundary conditions were only applied to the X and Z directions, keeping the Y direction free, in order to simulate actual modification at the atomic level. The REBO potential was used to characterize how C-C bonds hybridize [24].

The as-prepared diamond model was relaxed to its equilibrium configuration within the isothermal–isobaric NPT ensemble to ensure that the system was stable, by equilibrating at 300 K under 0 bar for 25 ps [22]. To protect the material from rigid movement during pulsed laser irradiation, the bottom of the workpiece, with a thickness of 2 Å, was fixed. The upper layers comprised the laser area shown by the arrow in Figure 2; the bottom layer comprised the thermostat. The MD simulation of pulsed laser modification consisted of laser irradiation and subsequent energy dissipation, and the laser modification was carried out by the deposition of kinetic energy. Laser irradiation involves depositing kinetic energy directly onto the surface atoms over a pulse duration of 65 ps. This process simulates the effect of a laser pulse by transferring kinetic energy to atoms within the irradiated area. In this study, the timestep was 0.0005 ps, the irradiation time of laser energy was 65 ps, and the energy diffusion time was 25 ps to 45 ps. 

The coordination number in crystallography is the quantity of nearby atoms that are within a given cutoff distance from the central atom. It is related to the crystal structure or cell type, and the two most popular coordinations in the model are fourfold and threefold coordination, which correspond to diamond and graphite structures, respectively. The major element of diamond is carbon, and it can form both sp^3^ and sp^2^ hybridized electronic states. In this article, to observe the change in diamond structure during the laser modification more clearly, atoms were colored according to their coordination numbers: black stood for atoms with a coordination number of 0, gray stood for atoms with a coordination number of 1, blue stood for atoms composing GBs and atoms with a coordination number of 3, light blue and red stood for atoms with coordination numbers of 2 and 4, respectively, and the red atoms were perfect diamond structures. The atoms with coordination numbers from 0 to 2 were liquid or gas. For better observation, the model was sliced in the Y direction with a thickness of 1 nm by visualization software.

## 3. Results and Discussion

### 3.1. Morphology of the Diamond Under Laser Modification

**Figure 3:**
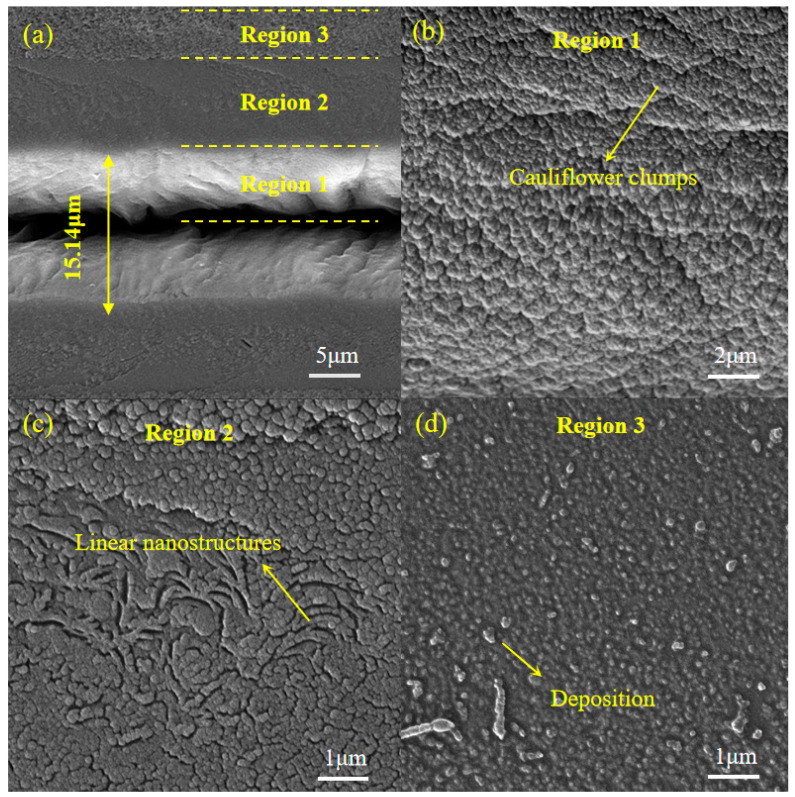
Morphology of the modified surface under a laser power of 50 mW. (**a**) Groove morphology; (**b**) Region 1; (**c**) Region 2; (**d**) Region 3.

Figure 3 shows the morphology of the CVD diamond under the laser modification with a laser power of 50 mW. As shown in Figure 3a, a microgroove with a width of 15.14 μm was fabricated on the laser modified region, which demonstrates obvious material removal. Figure 3b–d depict expanded views of region 1 to region 3, corresponding to the dotted lines in Figure 3a, indicating apparent debris depositions around and in the laser-modified region. In Figure 3b, large areas of cauliflower-like clumps were observed at the sidewall of the microgroove. Additionally, particles deposited outside the groove, as seen in Figure 3c,d, condensed into large cauliflower-like clumps and linear nanostructures. A clear boundary between region 2 and region 3 can be seen. Due to the energy distribution characteristics of the Gaussian beam, the energy in the center area of the microgroove is the highest, and the energy away from the groove gradually decreases. Therefore, the energies in region 2 and region 3 were different. Particles near to region 1 were fewer, it was likely that the high plasma plume pressure encouraged its early expansion in an ambient atmosphere and then sheltered this area from the deposition of debris particles [25]. There were many large debris particles in region 2, as shown in Figure 3c, which may be because of their excessive weight. 

**Figure 4:**
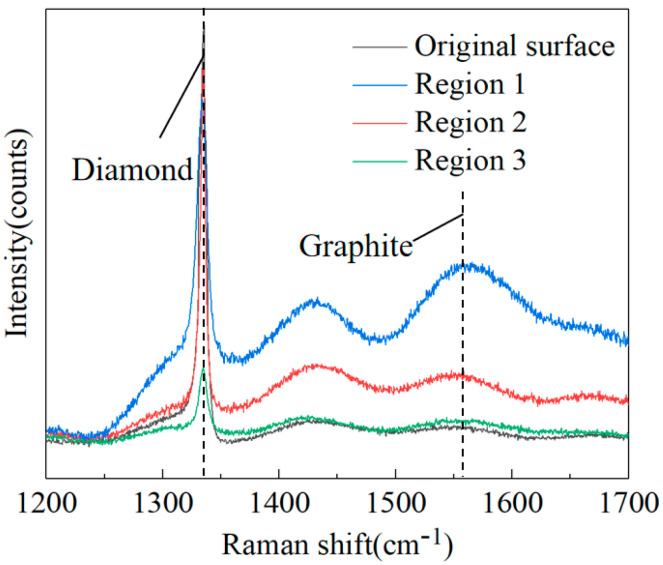
Raman spectra of the microstructure under a laser power of 50 mW.

Raman spectra are widely used to distinguish different allotropes of carbon, such as diamond and graphite. Raman spectra can also provide information in the comparison of diamond and graphite components [26]. Figure 4 shows the Raman spectra of four typical positions on the diamond surface under laser modification, corresponding to regions 1, 2, and 3 and the original surface, respectively. The spectrum of the original surface exhibits a strong narrow-band diamond peak at 1334 cm^−1^, which shows a frequency shift compared with natural diamond (1332 cm^−1^) [27], suggesting the presence of residual stress. For the laser-modified surface, the Raman spectra present two distinct peaks of the first-order diamond D-band at 1334 cm^−1^ and the first-order graphite G-band at 1543 cm^−1^, suggesting that the phase transition from diamond to graphite occurred. The peak at 1432 cm^−1^ indicates a phase transformation from diamond to sp^2^ allotropes. Moreover, Figure 4 demonstrates that the pronounced peaks of the sp^2^ carbon allotrope possess a wide full width at half maximum (FWHM), which can be attributed to the Raman active vibration mode of sp^2^ amorphous carbon [22,28]. The Raman spectra of microstructure at region 1 achieved a stronger graphite peak intensity, indicating greater graphite formation at the sidewall of the microgroove compared to other surrounding positions. The spectra of region 3 had the lowest graphite peak, indicating that there was almost no graphite in the region of material deposition.

**Figure 5:**
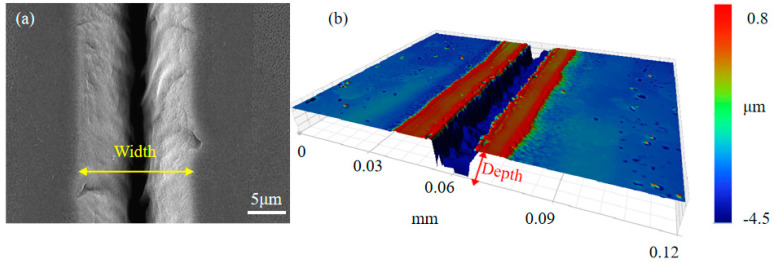
The morphology of the modification region under a laser power of 130 mW. (**a**) SEM image; (**b**) optical microscopy image.

The results of the laser modification of the diamond surface included a microgroove caused by material removal, the graphitization in the sidewall of microgroove and the deposits, and the morphology of each region being different due to the different energy distributions. Therefore, to research the effect of laser power on material removal and deposition, microgrooves on the surface were created using laser powers ranging from 80 mW to 130 mW. Figure 5a shows the nanodebris near the diamond microgroove under a laser power of 130 mW, which was less than that of 50 mW. This could be due to the increased laser energy density, which causes the rapid vaporization of the diamond material, resulting in less ablative debris being deposited on the diamond surface.

**Figure 6:**
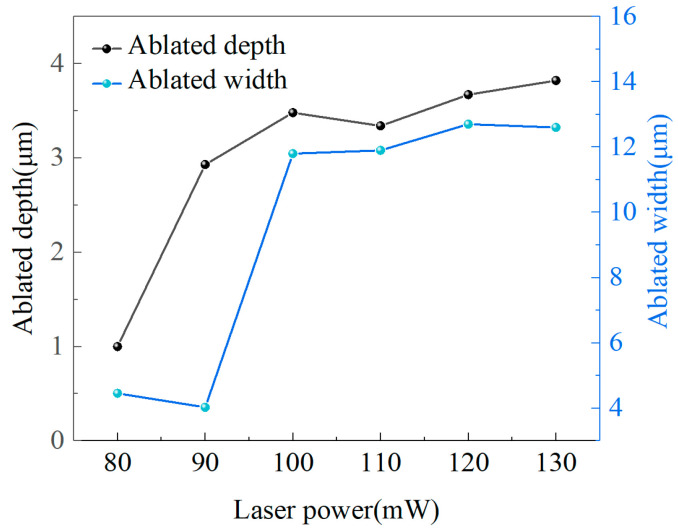
Ablated width and depth of the modification region under laser powers of 80 mW to 30 mW.

Furthermore, the 3D morphology of the microgroove was measured by optical microscopy, as shown in Figure 5b. The height of the surface on either side of the groove was 0.18 μm, caused by layers of deposition formed by particles sputtered during laser modification, and the micro-groove was a “V” shape. The width and depth of microgrooves in the region of modification with a laser power of 80 mW to 130 mW were measured by optical microscopy. The width of the microgroove in Figure 5a is twice the width of region 1 in Figure 3. Figure 6 shows the relationship between the width and depth of the microgrooves and the laser power. The width increased gradually as the laser power increased, but the increase was gentler when the laser power was high. Since the logarithm of the material removal rate is dependent on laser power, which is related to the level of laser power, the depth of material removal changes more gradually at higher laser powers due to the logarithmic effect [29]. Material removal on the diamond surface was evident under laser powers of 80 mW to 130 mW, as the laser energy density significantly exceeded the graphitization threshold. Given the uncertain effect of laser power on graphitization, it is crucial to understand the formation mechanisms of graphitization. Therefore, developing an MD simulation to study the modification of diamond using a nanosecond pulsed laser is necessary.

### 3.2. Results of MD Simulation

#### 3.2.1. Evolution Characteristics of Diamond Under Laser Modification

**Figure 7:**
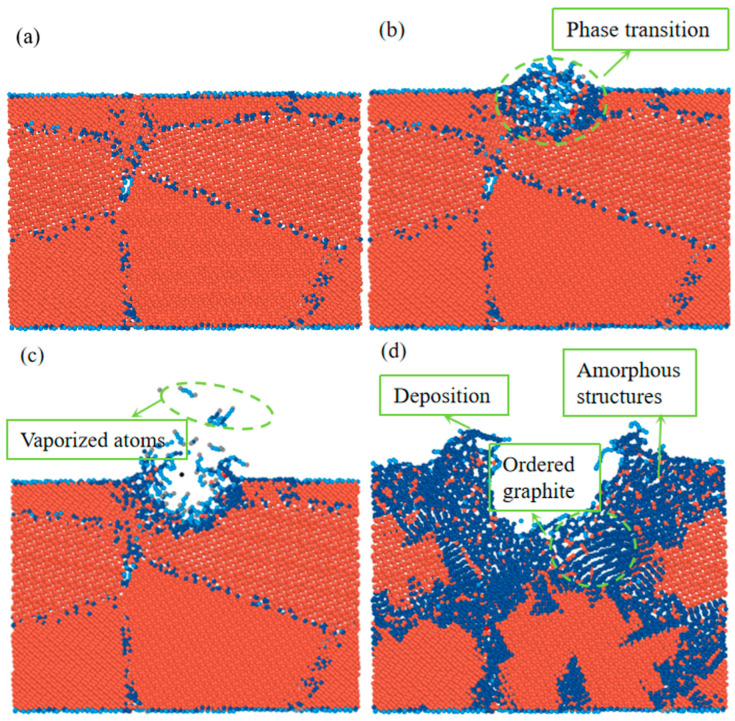
Results of the MD simulation at different times: (**a**) 0 ps, (**b**) 10 ps, (**c**) 20 ps, and (**d**) 65 ps.

The MD simulation of laser modification during 65 ps is shown in Figure 7. The polycrystalline diamond was converted into disordered amorphous carbon at the initial 10 ps of the injection of laser energy, as shown in Figure 7b, and it can be identified as graphite according to the coordination number [30]. In Figure 7c, we can see that phase transition accompanied by melting occurred on the surface, and atoms vaporized and escaped from the surface. Figure 7d shows that the material removal and graphitization increased with the increasing laser pulse time. Ultimately, after a 65 ps pulsed laser exposure, distinct disordered amorphous structures and ordered graphite structures were evident. These findings demonstrate that when the lattice was subjected to laser energy, the atoms moved out of their regular lattice positions, broke their bonds, and eventually created amorphous carbon and graphite. Graphite-layer atoms did not consistently occupy the same plane at different times; they originated from the hexagonal structures of the diamond lattice at various positions. These curved hexagonal structures in the diamond lattice gradually disintegrated, eventually forming the regular hexagons characteristic of the graphite lattice.

**Figure 8:**
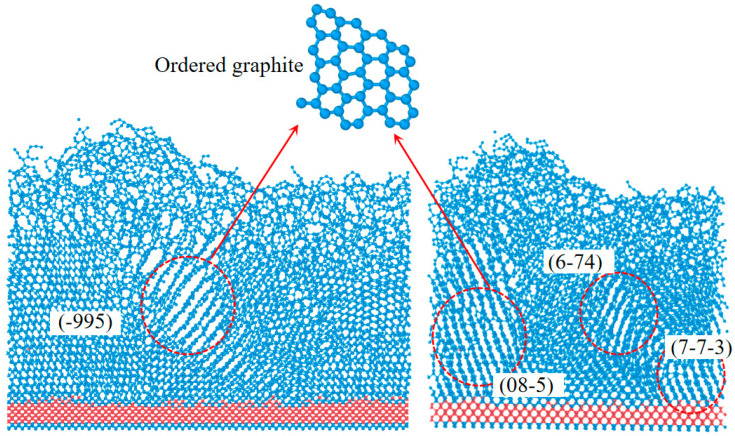
Results of MD simulation in diamond.

Figure 8 shows the MD simulation results for diamond after the energy gradually spread to the bottom of the model. Amorphous graphite and regular graphite layers existed simultaneously; graphite layers with different crystal orientations could be observed, as shown in the red circle, and their orientation could be determined. Regular layered graphite was mainly concentrated in the lower part of the model, and the upper part was amorphous. Several orientations of graphene such as (-995)/(08-5)/(6-74)/(7-7-3) were observed by slicing the model. In this study, none of the graphite adopted the usual crystal orientation of diamond, attributable to the random crystal orientation inherent in polycrystalline diamond.

**Figure 9:**
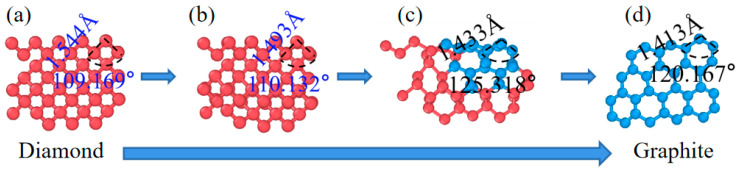
Evolution process from diamond to graphite.

A 2 nm slice of the model is extracted to understand the phase transition from diamond to graphite, as shown in Figure 9a–d. At the initial stage, the perfect diamond had four nearest neighbors, and its bond length and bond angle were 1.54 Å and 109°, respectively. As the laser energy spread, the bond length and bond angle evolved, as depicted in Figure 9b–d. The potential energy of the atoms in the perfect diamond was −7.37 eV, and the average potential energy of the atoms from Figure 9b–d shifted from −7.37 eV to −6.8 eV. It should be noted that the newly formed graphite lattice deviated from the structure of standard graphite; the interlayer distance ranged from 0.20 nm to 0.26 nm, contrasting with the standard graphite layer distance of 0.335 nm. Variations in potential energy were observed between different layer arrangements and among atoms within the same graphite layer, ranging from −6.57 eV to −7.1 eV. This disordered graphite lattice can be attributed to further relaxation, which is most likely only explicable by extremely large MD super cells [31]. 

**Figure 10:**
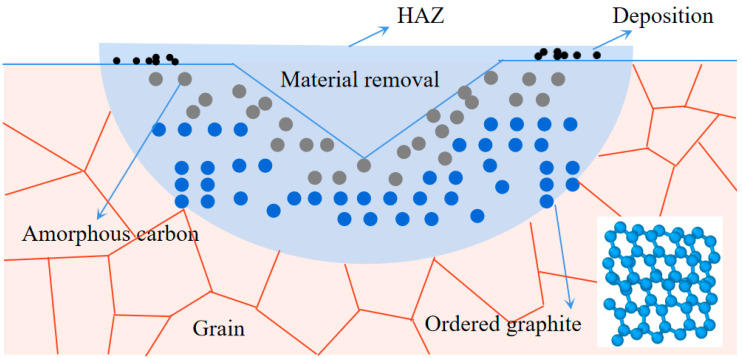
Result of laser graphitization in diamond.

Experimental and molecular dynamics (MD) simulation results have unveiled the mechanism behind laser-induced diamond modification: when a pulsed laser is applied and its intensity exceeds a certain threshold, the nonlinear interaction begins. The lower intrinsic absorption, resulting from interband transitions and point defects, leads the diamond’s laser-affected region to swiftly attain the critical temperature necessary for graphitization. Concurrently, this elevated temperature triggers the formation and growth of graphite nuclei. Therefore, under laser energy modification, the atoms in diamond transitioned from sp^3^ to sp^2^ hybridization. The resulting heat-affected zone (HAZ) in diamond encompassed material removal, material deposition, amorphous carbon, and regular graphite layer structures, as illustrated in Figure 10.

#### 3.2.2. Effect of Grain Boundaries on Graphitization

**Figure 11:**
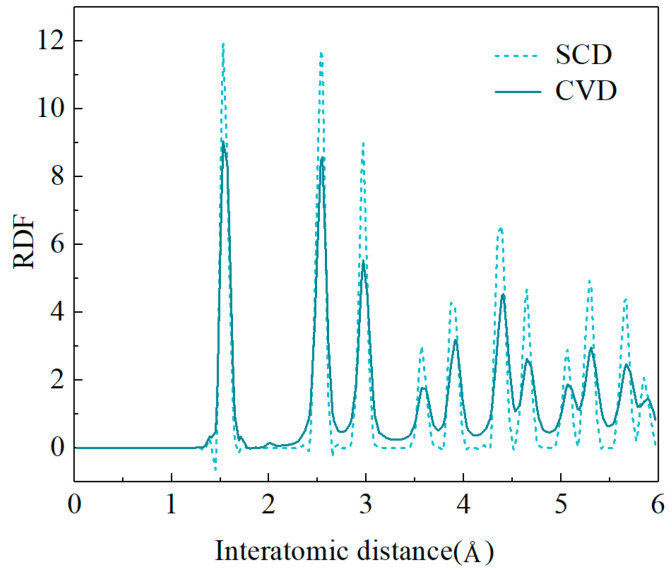
RDF of SCD and CVD diamond after relaxation.

Figure 11 shows the radial distribution function (RDF) of single-crystal diamond (SCD) and CVD diamond after relaxation. Differences in the peaks at long-range distances were observed, attributed to the loss of correlation in atomic positions when transitioning from one grain to another [32]. Although the peak values of CVD diamond and SCD were different, the widths of the peaks were similar at 300 K. This similarity indicated that polycrystalline CVD diamond, composed of multiple SCD grains and GBs, therefore, had similar properties to SCD.

**Figure 12:**
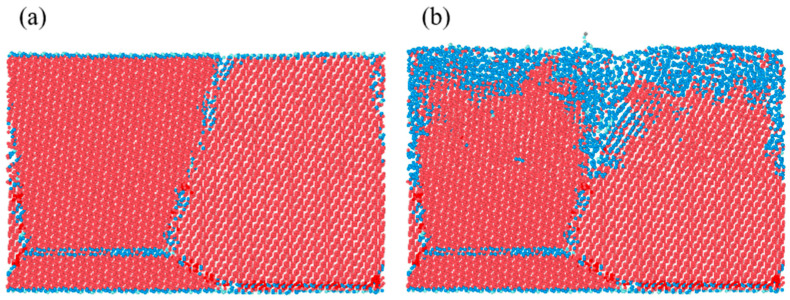
Results for polycrystalline diamond with two grains: (**a**) 0 ps and (**b**) 12.5 ps.

A model of polycrystalline diamond with two grains was established to research the effect of GBs on laser graphitization. In this model, atoms were colored gray, light blue, blue, or red according to their coordination numbers, which ranged from 1 to 4. Figure 12 shows the results for polycrystalline diamond with two grains. The crystal grains decreased, and the grain boundary was wider after 12.5 ps. The phase transition at the grain boundary between the two grains occurred earlier than within the grains themselves, as revealed by coordination number analysis. This is attributed to the higher potential energy of grain boundary atoms, ranging from −5 eV to −6 eV, compared to the standard diamond potential energy of −7.37 eV. This suggests that the presence of GBs plays a role in the graphitization of CVD diamond during laser modification. 

#### 3.2.3. Effect of Laser Power on Graphitization

**Figure 13:**
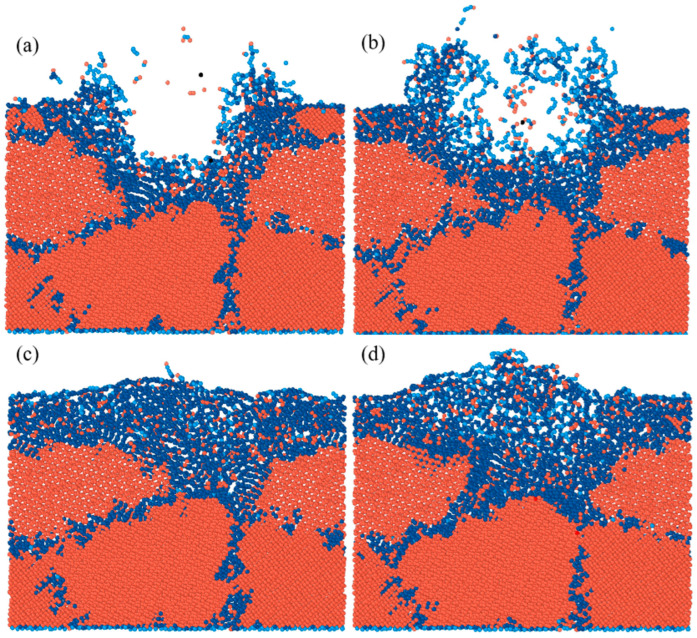
MD simulation of diamond under different laser spot radii after 40ps: (**a**) r = 15 Å, (**b**) r = 30 Å, (**c**) r = 45 Å, (**d**) r = 60 Å.

**Figure 14:**
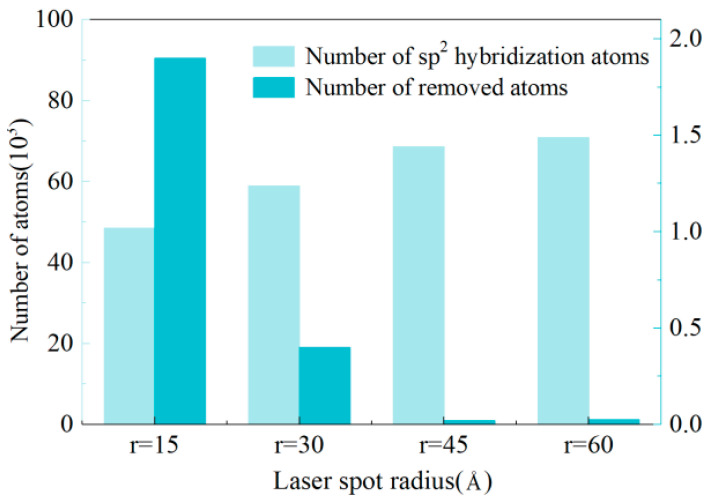
Number of sp^2^ hybridization atoms and number of removed atoms in diamond under different laser spot radii.

Four models were established to investigate the effect of laser power on graphitization, and the laser power was adjusted by changing the radius while fixing the laser energy value; in other words, the larger the radius of the laser heat region, the lower the laser power. An MD model with five grains was established, and the average grain size and grain boundary volume fraction of the as-prepared polycrystalline specimen were 230.4 nm^3^ and 24.9%, respectively. Figure 13 is the result of the MD simulation after 40 ps. In contrast to the results in Figure 13a,b, Figure 13c,d show a greater proportion of graphitization compared to material removal. The graphite structure consists of sp^2^ hybridized carbon. Figure 14 calculates the number of atoms removed (with coordination numbers of 0, 1, or 2) and the increased number of sp^2^ hybridized atoms (with a coordination number of 3) to compare graphitization under varying levels of laser power. The result distinctly indicates that the number of sp^2^ hybridization atoms increased with decreasing laser power. Notably, in Figure 13c,d, almost no material removal occurred, and the model changes were predominantly phase transitions. Therefore, it can be predicted that a smaller laser power can cause a higher proportion of graphitization to material removal. However, it is important to note that a laser power that is too low may not induce a significant phase transition. Therefore, it is necessary to carry out an experiment to study the effect of laser power on graphitization.

### 3.3. Results of Surface Modification and Precision Grinding in Diamond

The removal of diamond material during laser modification is divided into two steps: first, the diamond is graphitized, and then the graphite component is sublimed to achieve the removal of the material [29]. To achieve greater graphitization of CVD diamond compared to material removal, it is essential to investigate the laser ablation threshold of CVD diamond prior to conducting graphitization experiments. The laser ablation threshold of materials refers to the critical laser energy density required for the irreversible removal of materials due to high energy density during laser processing. The ablation threshold for a laser with a Gaussian profile can be determined using the method outlined below [33].

(1) $$D^{2}=2\omega_{0}^{2}\ln\frac{2p}{\pi \omega_{0}^{2}fF_{\mathrm{th}}}$$

where *D* is the diameter of the material removal, measured by SEM. *ω*_0_ is the focus radius, *P* is the average laser power, *f* is the laser repetition frequency, and *F*_th_ is the ablation threshold of the material.

By fitting the square of the hole diameter obtained by ablation and LnP, the slope k can be obtained, and the focus radius can be determined by slope k, and then the laser power when the diameter of the ablation hole is 0 can be obtained to obtain the laser ablation threshold.

When the diameter of the material removal is 0, the laser output power is

(2) $$P_{0}=\frac{1}{2}\pi \omega_{0}^{2}fF_{\mathrm{th}}$$

**Figure 15:**
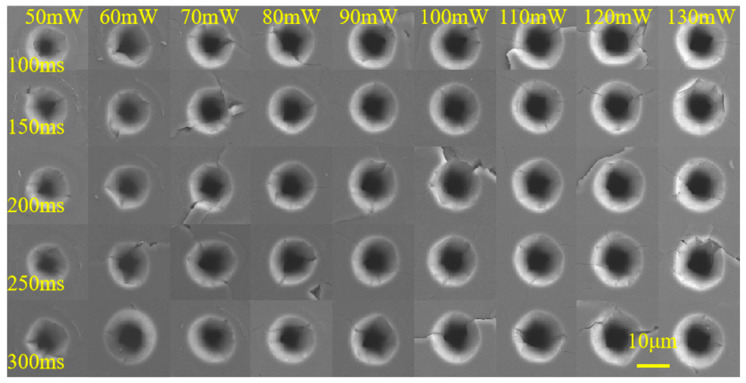
Results for the laser ablation threshold of the CVD diamond.

The laser power was 50 mW to 130 mW and the time was 100 ms to 300 ms. The laser pulse repetition frequency was *f* = 5 kHz. A laser ablation threshold experiment was carried out on the polished CVD diamond, and the results are shown in Figure 15. 

The experimental parameters and aperture measurement results indicate that the diameter of the laser-machined hole expands with increased laser processing time but contracts as the laser power decreases.

**Figure 16:**
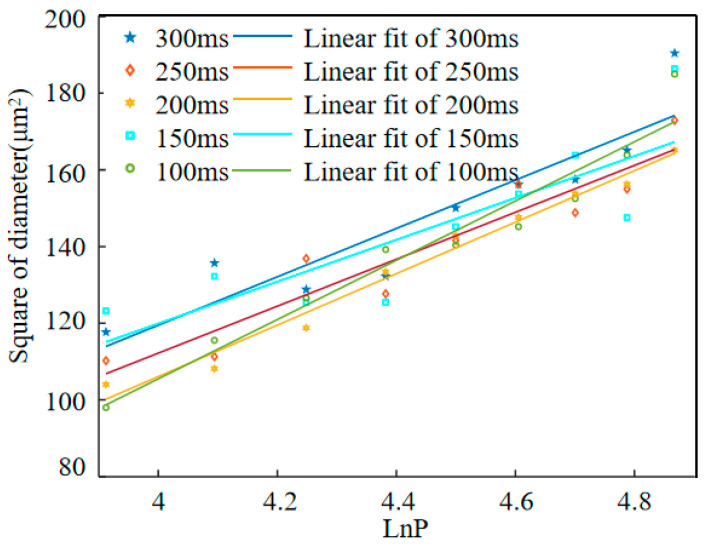
The relation between the diameter and laser power.

Based on the calculations in Equations (1) and (2), the relation between the diameter and logarithm power under different laser pulse numbers can be obtained, as shown in Figure 16. Then, the slope of the fitting line *k*, waist radius *ω*_0_, power at 0 ablation diameter *P*_0_, and laser ablation threshold for different action times can be obtained, as shown in Table 1.

**Figure 17:**
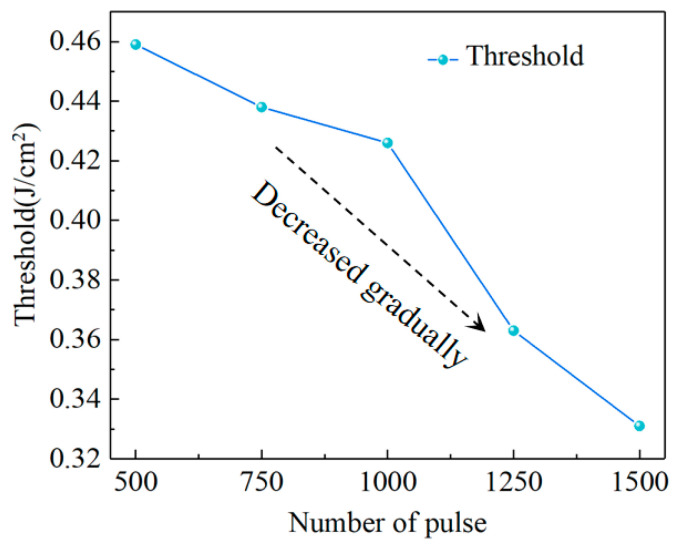
The relationship between the ablation threshold and the number of laser pulses.

Figure 17 illustrates the relationship between the diamond material ablation threshold and the number of laser pulses. Due to the cumulative effect of laser machining, the removal threshold gradually decreased with the increasing cumulative pulse number.

**Figure 18:**
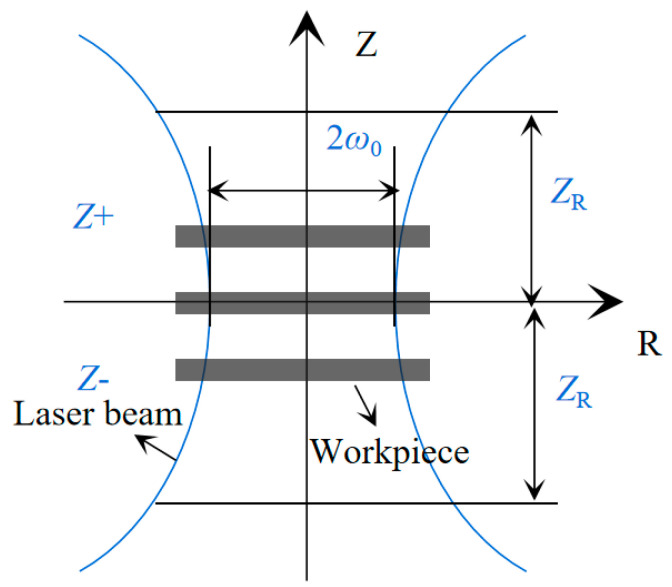
Schematic diagram of laser propagation.

Therefore, to achieve greater graphitization and reduced material removal in the graphitization experiment with CVD diamond, the laser power should be set close to the ablation threshold. The laser power can be adjusted by varying the defocusing quantity of the incident laser beam. Figure 18 shows a focused Gaussian laser beam. The laser spot radius at defocusing position Z can be calculated using Equation (3).

(3) $$\omega_{z}=\omega_{0}{\left[1+{\left(\frac{Z}{Z_{R}}\right)}^{2}\right]}^{\frac{1}{2}}$$

where *Z*_R_ is the Rayleigh length.

During the laser scanning experiment, the total number of laser pulses or equivalent pulses impacting a specific point along the scanning path can be calculated using Equation (4) [33].

(4) $$N=\frac{2\omega_{z}f_{p}}{v}$$

where *v* is the velocity of scanning, which can be obtained by Equation (4) and the given laser pulse. 

**Figure 19:**
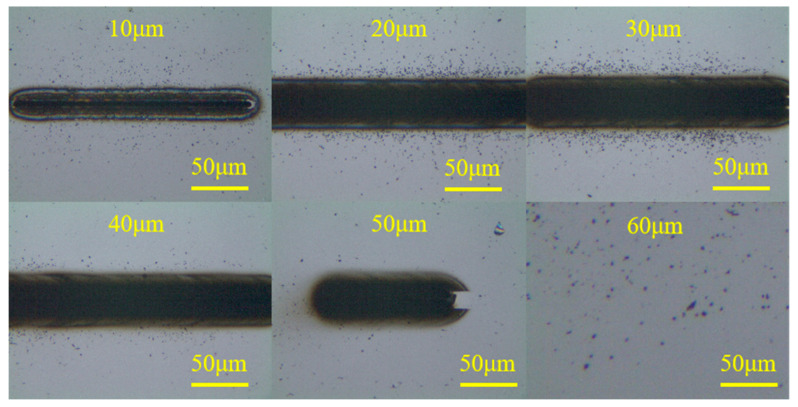
Modified surface with defocusing quantities from 10 μm to 60 μm.

**Figure 20:**
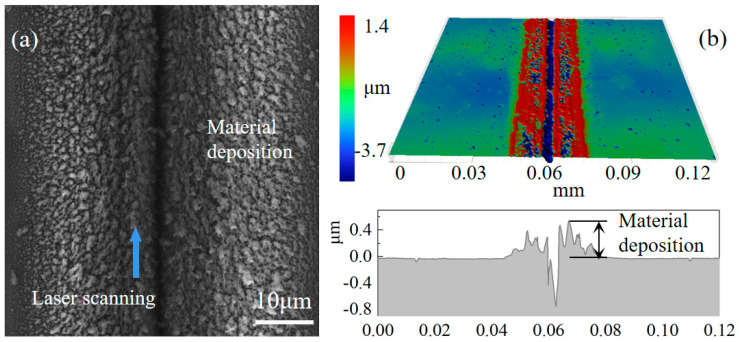
Result of defocusing quantity of 10 μm: (**a**) SEM image, (**b**) optical microscope image.

The focus points above 0 μm were examined in this work since there was no noticeable difference in ablation degree for modification with either negative or positive defocusing quantities. Thus, in order to research the effect of the defocusing position on laser graphitization, modified grooves with defocusing quantities from 10 μm to 60 μm were obtained, as presented in Figure 19. The laser scanning speed was 0.069 mm/s. A laser power exceeding the ablation threshold was used to ensure a clear ablative phenomenon. Distinct microgrooves were still observable at a defocusing quantity of 10 μm, indicating material removal. The width of the modified region increased most significantly from 10 μm to 20 μm but was not obvious between 20 μm and 50 μm. At a defocusing quantity of 60 μm, the surface showed no significant changes due to insufficient laser energy density. Figure 20 shows the results of SEM and optical microscopy. It is evident that significant material deposition occurred outside the microgrooves, consistent with the outcomes of MD simulations. The deposition layer likely formed because the laser power on the diamond surface decreased with increasing defocusing quantity, preventing complete material evaporation.

**Figure 21:**
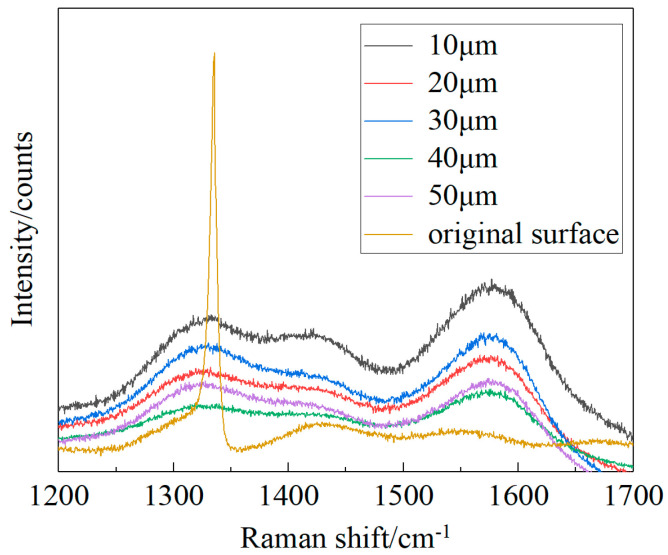
Raman spectra of the surface modified at different defocusing quantities.

**Figure 22:**
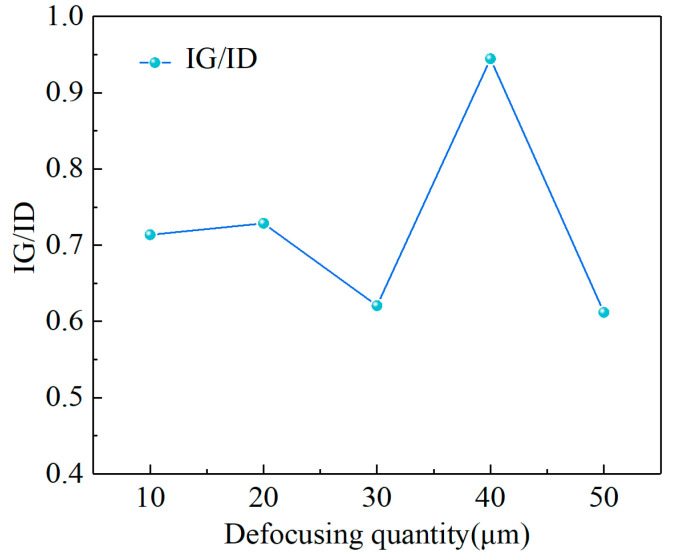
IG/ID at different defocusing quantities.

Figure 21 shows the Raman spectra of the modified region. The sp^2^ content can be reflected in the intensity ratio of graphite to diamond (IG/ID) [34]. The IG/ID ratio changed as the defocusing quantity increased from 10 μm to 50 μm, as shown in Figure 22. This suggested that more graphite was obtained on surfaces with a defocusing quantity of 40 μm. Therefore, in this work, a reduction in the material removal of CVD diamond and an increase in graphitization during laser modification could be achieved by changing the quantity of defocusing.

**Figure 23:**
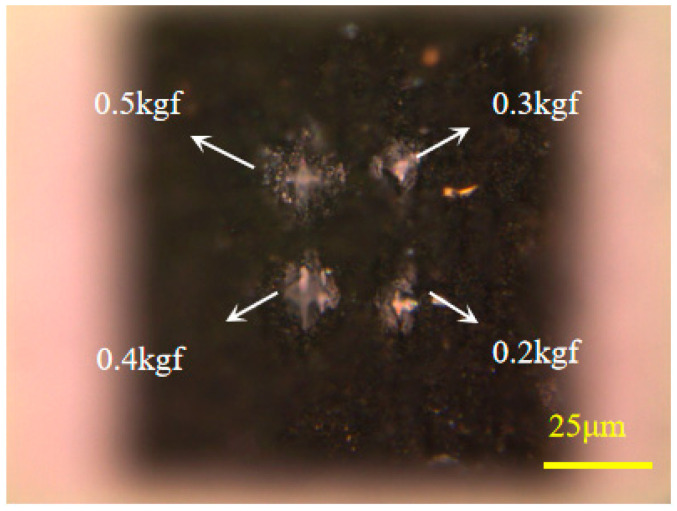
Indentation under different loads.

The surface hardness of diamond before and after modification was measured by a Vickers indentation instrument; the indentation shapes under various loads within the modified area are depicted in Figure 23, and 0.5 kgf was selected as a suitable load. The hardness was measured at different locations in the modified area by the Vickers indentation instrument. To minimize accidental errors and enhance the accuracy of the Vickers hardness measurements in the modified area, loads were applied at multiple positions on the cleaned modified surface, and measurements were conducted three times under the specified load. 

**Figure 24:**
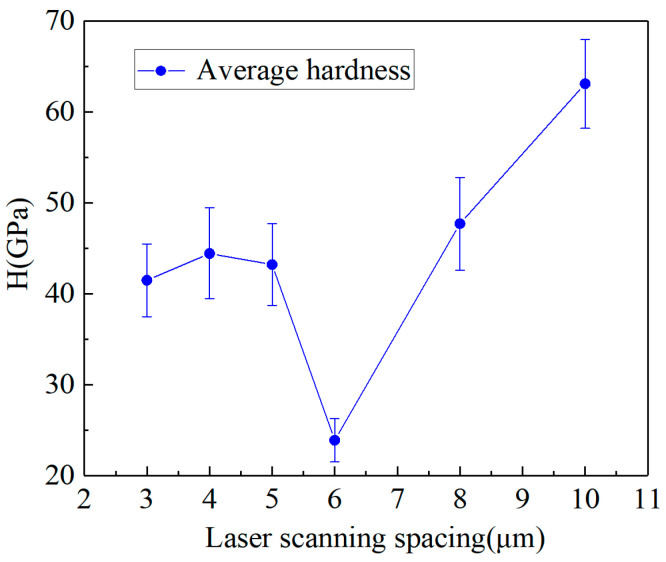
The average hardness of the modified surface obtained at different scanning spacings.

Figure 24 displays the average hardness of the modified surface, obtained using various laser scanning spacings under a defocusing quantity of 40 μm. The results indicated that the average hardness of the modified surface, ranging from 23.897 GPa to 63.104 GPa, was lower than that of diamond, which ranges from 70 GPa to 100 GPa. It can be observed from the figure that at a laser scanning distance of 6 μm, the hardness of the modified area reaches its lowest point. With a small scanning spacing, such as 3/4/5 micro, the scan overlap rate was high, and the modified area produced more material removal and less graphitization, so the hardness of the modified area was high; when the laser scanning was 6 μm, better control over material removal and graphitization was achieved, resulting in a quite obvious decrease in hardness. As the scanning distance increased beyond 6 μm, the surface hardness of the modified area increased. This is due to the scanning trajectory in these areas being composed of multi-channel micro-grooves, which have a low overlap rate, resulting in a non-uniform modified area. This suggests that the graphitization of CVD diamond induced by a nanosecond pulsed laser can reduce the material’s hardness and enhance its machinability.

**Figure 25:**
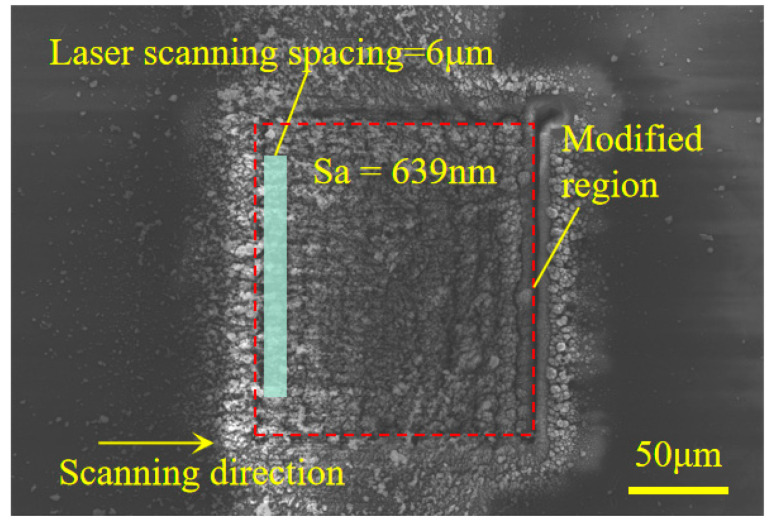
Image of the modified CVD diamond surface.

**Figure 26:**
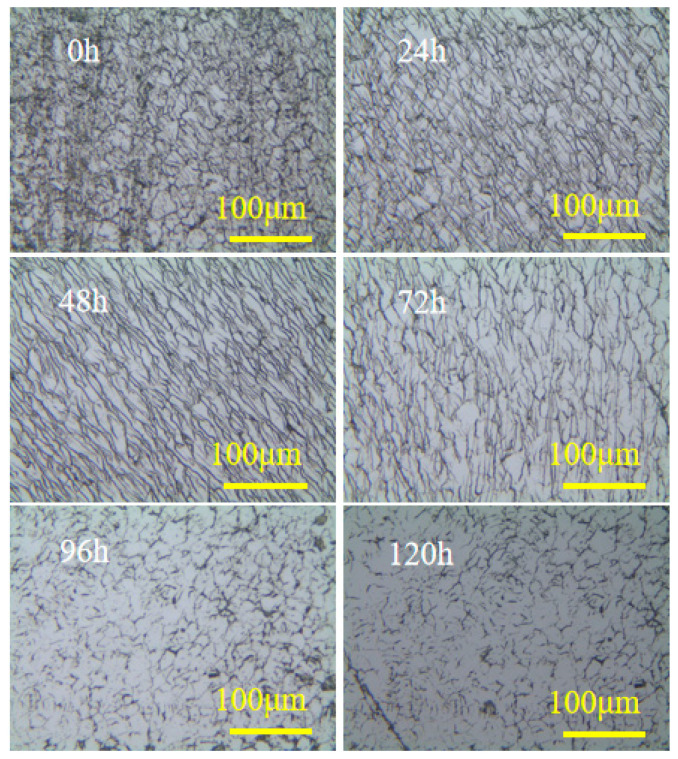
Morphology of the unmodified surface during precision grinding.

**Figure 27:**
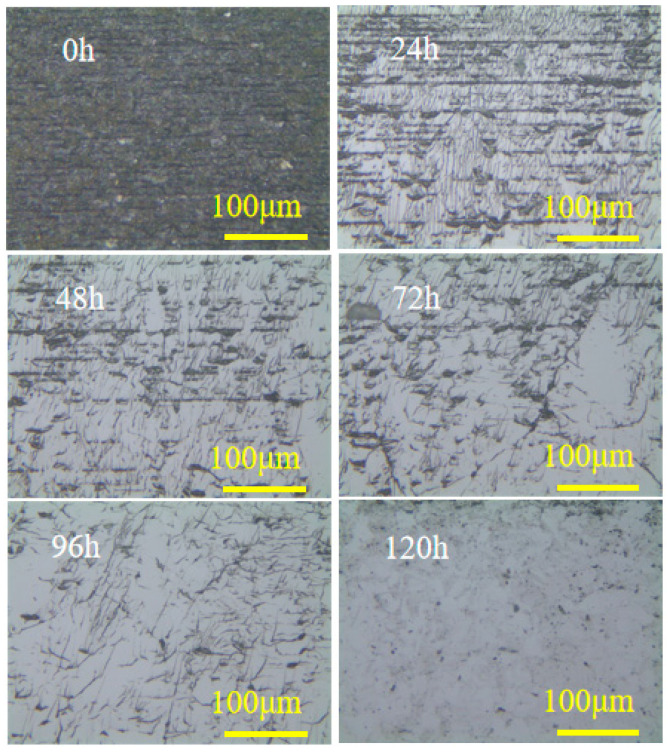
Morphology of the modified surface during precision grinding.

The modified CVD diamond surface was fabricated using a parallel nanosecond pulsed laser, with optimized graphitization parameters: frequency (f) = 5 kHz, scanning speed (v) = 0.069 mm/s, power (P) = 16 mW, defocusing quantity = 40 μm, and laser scanning spacing = 6 μm. Figure 25 displays the image of the modified CVD surface, which had a roughness of Sa = 639 nm. Figure 26 displays the surface morphology during the precision grinding process for both the modified and unmodified CVD diamond surfaces. The results indicate that the scratches on the unmodified surface were prominent at the 0-hour mark, and the surface roughness gradually decreased as the grinding time increased. After 120 hours, there were still scratches on the surface. Figure 27 shows that the modified surface remained black at the 0-hour mark due to the laser modification, with the parallel scanning trajectory visible. The black modified area was noticeably removed after 24 hours, and surface quality was significantly improved after 72 hours, with only grinding-induced scratches remaining on the surface. Ultimately, the surface became smooth after 120 hours.

**Figure 28:**
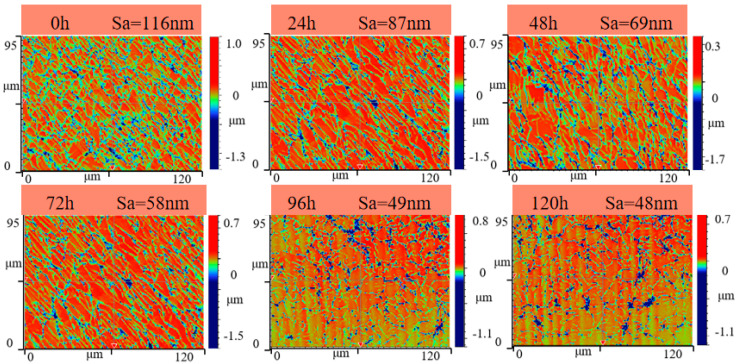
The 3D morphology of the unmodified surface.

**Figure 29:**
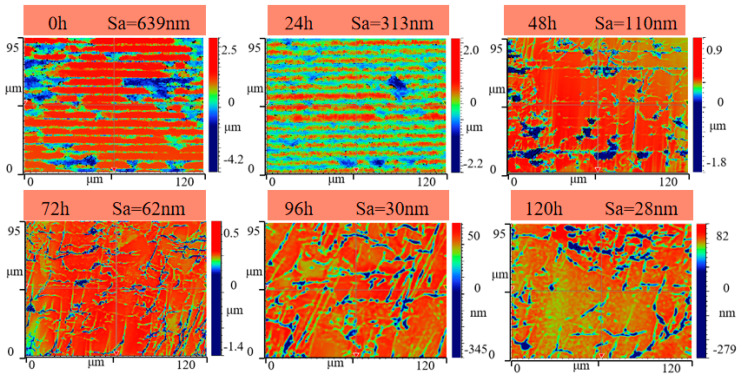
The 3D morphology of the modified surface.

Further studies were conducted on the roughness Sa during the precision grinding process for both the unmodified and modified surfaces of CVD diamond, as depicted in Figure 28 and Figure 29. In Figure 28, at the end of the precision grinding, the roughness Sa of the unmodified surface was finally reduced to 48 nm. Figure 29 shows that at the initial stage of grinding, the grooves and undulations in the modified surface, created by the laser scanning of the CVD diamond, remained clearly visible. After 48 hours, the quality of the modified surface had significantly improved, and the surface roughness Sa gradually decreased to 110 nm, matching the level of the unmodified diamond at the 0-hour mark in the comparative experiment. The surface roughness Sa of the modified surface was further reduced to 28 nm after 120 hours.

**Figure 30:**
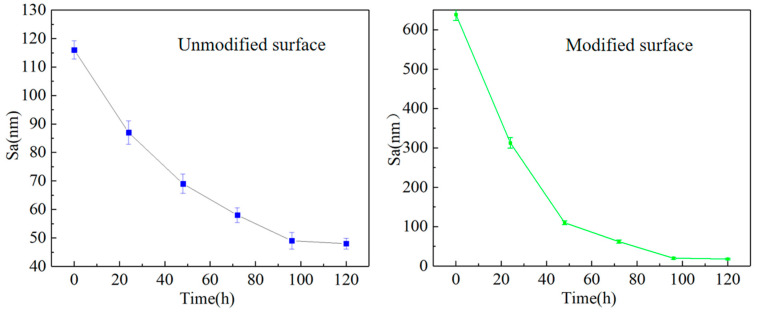
The variation in the unmodified surface and modified surface roughness Sa with time.

Figure 30 illustrates the variation in roughness Sa for both the unmodified and modified surfaces of CVD diamond over the course of precision grinding. At the outset of the experiment, the modified surface’s roughness dropped rapidly from 639 nm to 110 nm within the first 48 hours, matching the 116 nm roughness Sa of the unmodified surface at the 0-hour mark. After 96 hours, the decline trend was gradual, and Sa finally reached 28 nm. The surface roughness of the unmodified CVD diamond decreased from 116 nm to 48 nm within 120 hours. Consequently, under identical experimental conditions, the unmodified CVD diamond exhibited a higher surface roughness Sa compared to the modified area, indicating a reduced efficiency in precision grinding

## 4. Conclusions

In this research, the characteristics of graphitization during the modification of diamond using a nanosecond pulsed laser were investigated. Based on the MD simulation and laser modification experiments, the main conclusions can be summarized as follows:

1. The analysis of the diamond’s modified surface after pulsed laser treatment revealed that the main characteristics of laser modification for polycrystalline diamond include microgrooves caused by material removal, the graphitization on the sidewalls of microgrooves and in deposits, and variations in each region’s morphology due to distinct energy distributions.
2. Besides material removal, the laser modification process also led to the formation of disordered amorphous structures and distinct lamellar graphite structures. Concurrently, atoms that evaporated and escaped from the diamond surface were deposited back onto it, a finding consistent with the experimental outcomes of laser modification. The grain boundaries in polycrystalline diamond were confirmed to contribute to graphitization during laser modification. Furthermore, the orientations of the graphite are likely a reflection of the random grain orientations within the polycrystalline diamond.
3. An increased graphitization rate can be achieved by increasing the defocusing distance while maintaining a fixed laser power. In grinding experiments, the surface roughness Sa of CVD diamond post-optimized laser modification was significantly reduced, with a higher reduction rate compared to the unmodified CVD diamond matrix. The experimental results confirmed the effectiveness of laser graphitization pretreatment in enhancing the efficiency and quality of subsequent grinding processes.

## Figures and Tables

**Table 1:** The calculation results for different numbers of laser pulses.

Numbers of Pulses	*k*	*ω*_0_ (μm)	*P*_0_ (mW)	Threshold (J/cm^2^)
1500	63.0258	5.6136	8.196	0.331
1250	61.1819	5.5309	8.720	0.363
1000	67.1368	5.7938	11.242	0.426
750	54.5851	5.2242	6.060	0.438
500	77.2765	6.2160	13.936	0.459

## Data Availability

The original contributions presented in this study are included in the article. Further inquiries can be directed to the corresponding author.
